# Abnormal Neural Processing during Emotional Salience Attribution of Affective Asymmetry in Patients with Schizophrenia

**DOI:** 10.1371/journal.pone.0090792

**Published:** 2014-03-11

**Authors:** Seon-Koo Lee, Ji Won Chun, Jung Suk Lee, Hae-Jeong Park, Young-Chul Jung, Jeong-Ho Seok, Jae-Jin Kim

**Affiliations:** 1 Institute of Behavioral Science in Medicine, Yonsei University College of Medicine, Seoul, Republic of Korea; 2 Department of Psychiatry, National Health Insurance Ilsan Hospital, Koyang Gyeonggi, Republic of Korea; 3 Department of Psychiatry, Bundang Jesaeng Hospital, Seongnam, Republic of Korea; 4 Department of Radiology, Yonsei University College of Medicine, Seoul, Republic of Korea; 5 Department of Psychiatry, Yonsei University College of Medicine, Seoul, Republic of Korea; University of Colorado Medical School, United States of America

## Abstract

Aberrant emotional salience attribution has been reported to be an important clinical feature in patients with schizophrenia. Real life stimuli that incorporate both positive and negative emotional traits lead to affective asymmetry such as negativity bias and positivity offset. In this study, we investigated the neural correlates of emotional salience attribution in patients with schizophrenia when affective asymmetry was processed. Fifteen patients with schizophrenia and 14 healthy controls were scanned using functional magnetic resonance imaging (fMRI) while performing an emotion judgment task in which two pictures were juxtaposed. The task consisted of responding to affective asymmetry condition (ambivalent and neutral) and affective symmetry conditions (positive and negative), and group comparisons were performed for each condition. Significantly higher activity in the medial prefrontal cortex and inferior frontal gyrus was observed for the ambivalent condition than for the other conditions in controls, but not in patients. Compared with controls, patients showed decreased activities in the dorsolateral prefrontal cortex, dorsal anterior cingulate cortex, insula, and putamen for the ambivalent condition, but no changes were observed for the neutral condition. Multiple prefrontal hypoactivities during salience attribution of negativity bias in schizophrenia may underlie deficits in the integrative processing of emotional information. Regional abnormalities in the salience network may be the basis of defective emotional salience attribution in schizophrenia, which is likely involved in symptom formation and social dysfunction.

## Introduction

Salience refers to the state or quality of an item that makes it stand out relative to its neighbors [1]–[3]. Accurate appraisal of salience in the environment is central to organisms' survivals in that salience detection facilitates learning and survival by enabling organisms to focus their limited perceptual and cognitive resources on the most pertinent subset of the available sensory data [4]. Salience is also of great importance in social cognition [5]. If we cannot determine the salient message among the large amount of information that social interactions provide, we may misunderstand other's intentions or feelings and behave in an inappropriate manner.

It has been suggested that patients with schizophrenia experience a state of aberrant salience attribution [6]–[8], and that patients' attribution of incentive salience to irrelevant stimuli contributes to the formation of delusions or hallucinations [7]. Previous studies of aberrant salience attribution have shown that patients with schizophrenia tend to imbue emotionality to neutral stimuli. For example, compared to normal controls, patients with schizophrenia gave higher pleasantness and unpleasantness rating scores to neutral stimuli [9], [10], and showed more positive responses to neutral stimuli [11], [12]. In contrast, in face recognition tests, patients with schizophrenia felt more negative emotion towards neutral faces [13].

In studying the neural bases of aberrant salience attribution in patients with schizophrenia, several structures are of particular interest. First, the dorsal anterior cingulate cortex (ACC) and insula have been identified as a salience network that functions to recognize the most relevant of several stimuli in order to guide behavior [14]–[16]. Activities in the two regions were shown to be altered when patients with schizophrenia experienced hallucinations or delusions, which might be a consequence of emotional salience to mundane events [14], [17], [18]. Second, the mesolimbic system for dopaminergic signaling and reward anticipation contributes to salience attribution [19]. Salience attribution in schizophrenia has been associated with altered activities in the mesolimbic system including the striatum [20]–[22], amygdala [8], hippocampus [13], and parahippocampal gyrus [23]. Third, the ventrolateral prefrontal cortex (VLPFC) functions to integrate cognitive and motivational information to compute behavioral significance, which can be used for goal-directed behaviors [24], [25]. In a previous study, salience coding in schizophrenia induced decreased activity in the VLPFC, which was linked to anhedonia [26].

Real life situations are complex and can involve conflicting emotions, such as stimuli incorporating both positive and negative emotions together. When a person evaluates these complex stimuli, one emotion is disproportionately more influential in the holistic appraisal than the other emotion, producing affective asymmetry. The first example is “negativity bias,” which refers to a tendency for the negative system to respond more intensely than the positive system when evaluative input increases [27], [28]. This may aid in anticipating threatening situations and protecting from danger as soon as possible [28], [29]. The second example is “positivity offset,” which refers to a tendency for the positive system to respond more than the negative system when evaluative input is weak or absent [28], [29]. This may facilitate more active exploration of the environment [28].

A previous neuroimaging study performed by our research group [30] revealed that the dorsolateral prefrontal cortex (DLPFC) was involved in the processing of negativity bias and positivity offset, suggesting that affective asymmetry may be caused by integrative functions at the neocortical level. A behavioral study demonstrated that patients with schizophrenia processed affective asymmetry in an impaired manner [31]. Based on these findings, patients with schizophrenia could potentially show a deficit in dorsolateral prefrontal function during the processing of affective asymmetry, but this hypothesis has not been examined yet.

The aim of the current study was to investigate the neural correlates associated with emotional salience attribution in patients with schizophrenia when the positive and negative systems were co-activated with asymmetric manifestations. To explore these functional correlates, we used an emotion judgment task involving two pictures with similar or different emotions during event-related functional magnetic resonance imaging (fMRI). We made the following predictions based on previous findings. First, when two opposite emotions are induced simultaneously, both patients and controls will show a discrepancy in the degree of attention paid to each emotion (positive or negative), resulting in emotional salience attribution. Second, emotional salience attribution that affects the initial perception of stimuli with two opposite emotional traits will be associated with impaired processing of affective asymmetry in patients with schizophrenia. Third, when affective asymmetry is processed, compared with controls, patients will show altered activations in brain regions related to salience attribution, as well as affective asymmetry, such as the DLPFC, VLPFC, ACC, insula, striatum, amygdala, hippocampus, and parahippocampal gyrus.

## Materials and Methods

### Participants

Fifteen patients with schizophrenia (eight men) and 14 normal controls (six men) participated in this study. Exclusive diagnosis of schizophrenia in patients and exclusion of any psychiatric disorders in controls were made using the Structural Clinical Interview for DSM-IV [32]. Exclusion criteria included the presence of a neurological or significant medical illness, current or past substance abuse or dependence, and left-handedness. Paranoid tendency was assessed using the paranoia scale [33]. Ambivalence disposition was measured using the schizotypal ambivalence scale (SAS) [34]. Psychopathological symptoms were assessed using the positive and negative syndrome scale (PANSS) [35]. Demographic and clinical data are provided in Table 1. Our study was carried out under the protocols approved by the institutional review board of Yonsei University Severance Hospital, and written informed consent was obtained from all participants.

**Table 1:** Demographic and clinical characteristics of patients with schizophrenia and healthy controls.

	Schizophrenia (N = 15)		Control (N = 14)			
	8 (53.3%)		6 (42.9%)		X^2^/t	p
Male, n (%)	Mean	SD	Mean	SD	0.318	0.573
Age (years)	31.7	6.8	30.6	5.5	−0.471	0.64
Education (years)	13.7	1.7	16.1	2.4	3.228	0.003**
Duration of illness (years)	10.3	6.9	–	–	–	–
CP equivalent dose (mg)	489.1	521.7	–	–	–	–
SAS	7.3	5.3	3.9	3.1	−2.154	0.04*
Paranoia scale	48.0	18.6	38.8	6.2	−1.764	0.05*
PANSS_Total	56.5	12.1	–	–	–	–
PANSS_Positive	13.4	4.2	–	–	–	–
PANSS_Negative	15.8	5.3	–	–	–	–
PANSS_General	27.3	7.5	–	–	–	–

SD, Standard deviation; CP, Chlorpromazine; SAS, Schizotypal Ambivalence Scale; PANSS, Positive and Negative Syndrome Scale.

*p<0.05,

**p<0.01.

### Stimulus Materials and Experimental Task

**Figure 1:**
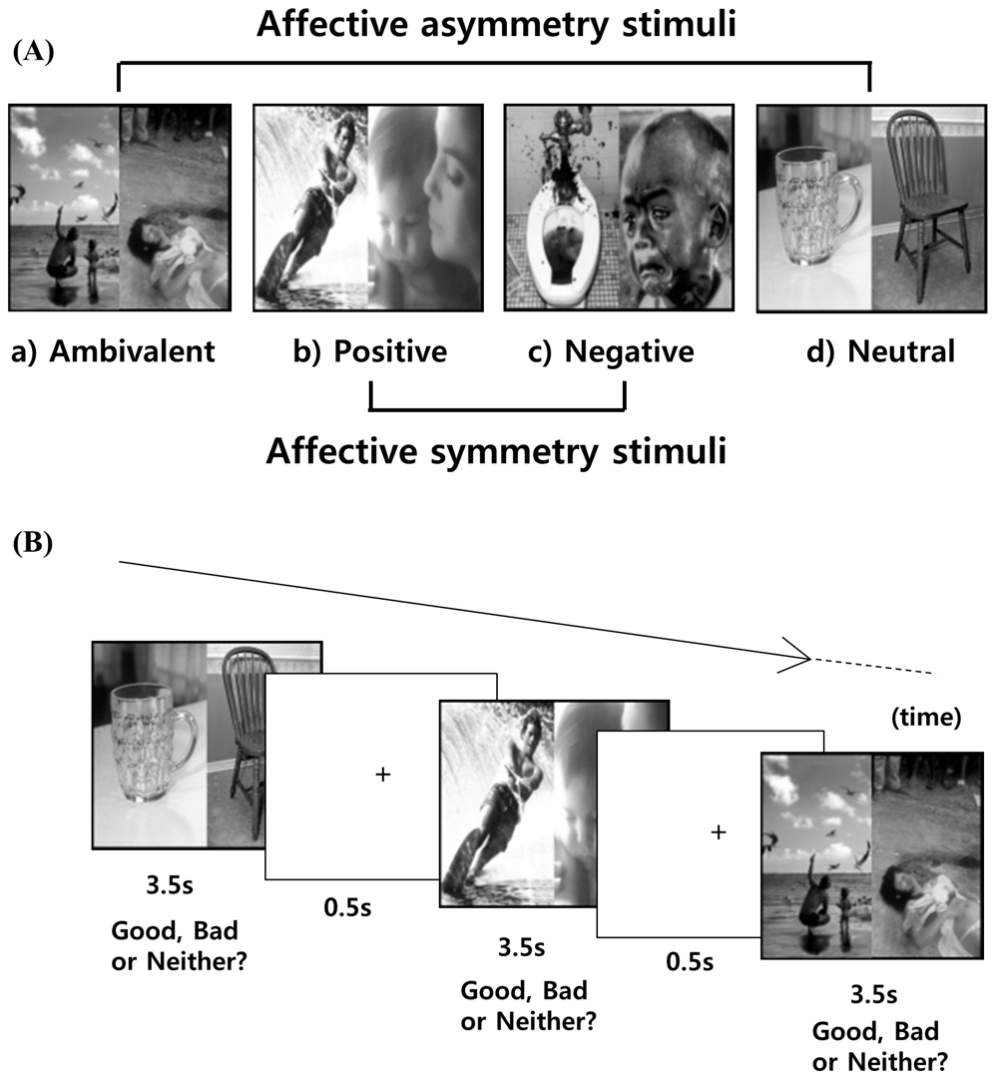
An example of the emotion judgment task.

During fMRI, participants performed an emotion judgment task, in which nine positive, nine negative, and nine neutral images (Table S1) from the International Affective Picture System [36] were used after modification (Figure 1); their mean valence was 7.53±0.18, 2.20±0.69, and 4.84±0.11, respectively. The two pictures juxtaposed represented four different conditions: ambivalent, positive, negative, and neutral, comprising pairs of positive-negative or negative-positive, positive-positive, negative-negative, and neutral-neutral images, respectively. A total of 160 pairs, with 40 pairs per condition, were presented in fully randomized order in an event-related design. All stimuli were presented for 3.5 seconds (ISI = 500 ms). The null events of crosshair fixation varied from 1.25 seconds to 10 seconds. Participants were encouraged to respond by clicking one of three buttons as quickly as possible, depending upon the subjective feeling produced by pairs of pictures as a unit. They could click the left, right, or middle mouse buttons to a positive, negative, or neither-positive-nor-negative (nPnN) response to the stimuli, respectively. Response types and reaction times were automatically transferred to the computer files (Table S2). Stimuli presentation and response recordings were performed using the software IFIS SA (MRI Devices Corporation, Waukesha, WI) and E-Prime system (Psychology Software Tools, Inc., Pittsburgh, PA).

### MRI Procedure and Image Preprocessing

MRI data were acquired on a 3T MR scanner (Intra Achieva; Philips Medical System, Best, Netherlands). Twenty-eight contiguous 4.5-mm-thick axial slices covering the entire brain were collected using a single-shot, T2*-weighted echo planar imaging sequence depicting the blood-oxygenation-level-dependent (BOLD) signal (echo time = 50 ms; repetition time = 2,000 ms; flip angle = 90°; field of view = 220 mm; and image matrix = 64×64). Axial 1.5-mm-thick T1-weighted images (echo time = 4.6 ms; repetition time = 25 ms; flip angle = 30°; field of view = 240 mm; and image matrix 256×256) were also collected.

Spatial preprocessing and statistical analyses were performed using Statistical Parametric Mapping 8 (SPM8). Corrections for differences in slice acquisition time were performed using user-specified sequences. Head motion was corrected by realignment, and corrected images were co-registered to the T1-weighted image for each participant. T1-weighted images were normalized to the standard T1 template, and the resulting transformation matrices were applied to the co-registered functional images. Normalized images were smoothed with an 8-mm full-width-at-half-maximum Gaussian filter.

### Statistical Analyses

#### Behavioral data analysis

Demographic and clinical data were compared between patients and controls by independent samples *t*-tests and chi-square tests. Response rates were analyzed using the Generalized Linear Mixed Model [37], in which each subject was considered to be a random effect and the factors were the group and emotional condition. The effect of the group and emotional condition on the reaction time was analyzed using analysis of covariance (ANCOVA), in which only total and the most frequent responses in each condition were included as a variable because of large differences among response rates. In all analyses, years of education were used as a covariate because they showed a significant group difference. The association between affective asymmetry including negativity bias and positivity offset and clinical measurement including SAS and paranoia scale was examined using regression analysis of General Linear Model (GLM), which was applied because there were individuals that showed response rates close to the extreme. In order to quantify affective asymmetry, negativity bias score was defined as the percentage of “negative responses” for the ambivalent condition in which evaluative input was increased, whereas the positivity offset score was calculated as the percent of “positive responses” for the neutral condition in which evaluative input was weak [30], [31].

#### Neuroimaging data analysis

Experimental trials for all emotional conditions and null trials were modeled separately for each condition minus the null events using a canonical hemodynamic response function and its first-order temporal derivative. The group and condition effects, as well as their interactions, were analyzed using ANOVA, and a two-sample *t*-test on the whole brain volume was performed for screening group comparisons. In these two analyses, education level was used as a covariate, and the threshold was set at an uncorrected *p*<0.001 with more than 30 contiguous voxels. Then, a two-sample *t*-test after small volume correction (SVC) with a threshold at a family-wise error-corrected *p*<0.05 was performed in the *a priori* regions, which were related to affective asymmetry and salience attribution. The investigated regions and their coordinates (x, y, z) included the DLPFC (−36, 36, 40; −36, 54, 6) [30], VLPFC (42, 24, −15) [26], dorsal ACC (6,22,30; −6,18,30) [15], insula (37, 25, −4; −32, 24, −6) [16], putamen (16, 12, 0; −16, 12, 0) [20], [21], amygdala (−21, −6, −12) [4], hippocampus (44, −24, −12) [13], and parahippocampal gyrus (18, −22, −18) [23]. The volume comprised a sphere with a 15-mm diameter for the DLPFC and VLPFC or 10-mm diameter for the other regions. For further analysis, percentage signal changes in significant clusters from this SVC analysis were obtained in each subject using MarsBaR version 0.41 (http://marsbar.sourceforge.net/). Correlations of regional percent signal changes with negativity bias and positivity offset scores were examined using regression analysis of GLM, in which *p*-values were adjusted for multiple correlations using a sequential Holm-Bonferroni procedure.

## Results

### Behavioral Data

As shown in Table 2, for the ambivalent condition, “negative” responses were the most frequent in both groups, and there were no significant differences in response types between the two groups. For the neutral condition, “nPnN” responses were the most frequent in both groups, but patients showed significantly higher “positive” and “negative” response rates and a significantly lower “nPnN” response rate than controls (p<0.001 in all comparisons). For the positive and negative conditions, the most frequent responses were “positive” and “negative” in both groups, respectively; however, “positive” and “negative” response rates were significantly lower in patients than in controls (p<0.001 in both comparisons). For all conditions, missing rates were significantly higher in patients than in controls (p<0.001 in all comparisons). The reaction times for total and the most frequent responses in each condition (Table S3) did not show a significant fixed effect for group and condition. In addition, there was no significant correlation between affective asymmetry scores and clinical measures.

**Table 2:** Group comparison of the response rates in each condition.

Condition	Response type	Response rate (%)		
		Schizophrenia (n = 15)	Control (n = 14)	p
Ambivalent	Missing	5.0 (7.8)	4.4 (5.4)	<0.0001*
	Positive	8.9 (12.3)	10.9 (8.2)	0.9828
	Negative	53.4 (31.2)	53.6 (33.8)	0.0435
	nPnN	32.7 (31.5)	31.1 (31.8)	0.6522
Positive	Missing	2.6 (6.3)	4.1 (5.2)	<0.0001*
	Positive	63.6 (26.3)	86.8 (14.1)	<0.0001*
	Negative	19.3 (22.5)	2.0 (3.7)	<0.0001*
	nPnN	14.5 (16.2)	7.1 (9.9)	0.0004*
Negative	Missing	1.8 (3.0)	5.2 (6.7)	<0.0001*
	Positive	7.5 (15.0)	2.1 (3.8)	<0.0001*
	Negative	81.6 (26.3)	91.4 (11.0)	<0.0001*
	nPnN	9.1 (14.7)	1.3 (2.4)	<0.0001*
Neutral	Missing	5.3 (9.5)	5.0 (6.4)	<0.0001*
	Positive	23.6 (19.4))	5.9 (6.8)	<0.0001*
	Negative	23.2 (26.8)	2.3 (2.7)	<0.0001*
	nPnN	47.9 (27.7)	86.8 (8.7)	<0.0001*

Data are given as means and standard deviation (SD). Group effects were testified using the Generalized Linear Mixed Model (GLMM) with years of education as a covariate.

*indicates significant difference (p<0.01) after Bonferroni correction.

“nPnN” means neither positive nor negative. Missing represents that participants did not respond.

### Imaging Data

**Figure 2:**
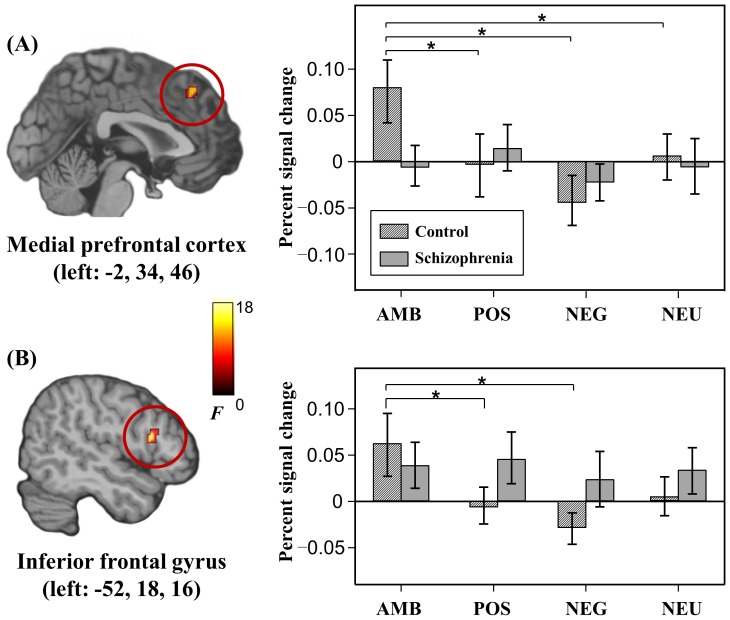
Brain regions showing significant group×condition interaction and percent signal changes of the regions for each condition. Error bar represents one standard error of the mean. * surpasses Holm-Bonferroni corrected threshold. AMB, ambivalent; POS, positive; NEG, negative; and NEU, neutral.

Brain regions showing a significant main effect for group and condition are summarized in Table S4. Significant group×condition interaction was found in the left medial prefrontal cortex and left inferior frontal gyrus (Figure 2). Post-hoc tests using regional percent signal changes revealed neither significant group difference in all conditions, nor significant condition difference in patients. In controls, however, there were significant condition differences: the ambivalent condition showed significantly higher percent signal changes than the negative (p<0.001), positive (p = 0.004), and neutral (p = 0.003) conditions in the left medial prefrontal cortex (p<0.001), as well as the negative (p = 0.002) and positive (p = 0.002) conditions in the left inferior frontal gyrus.

**Figure 3:**
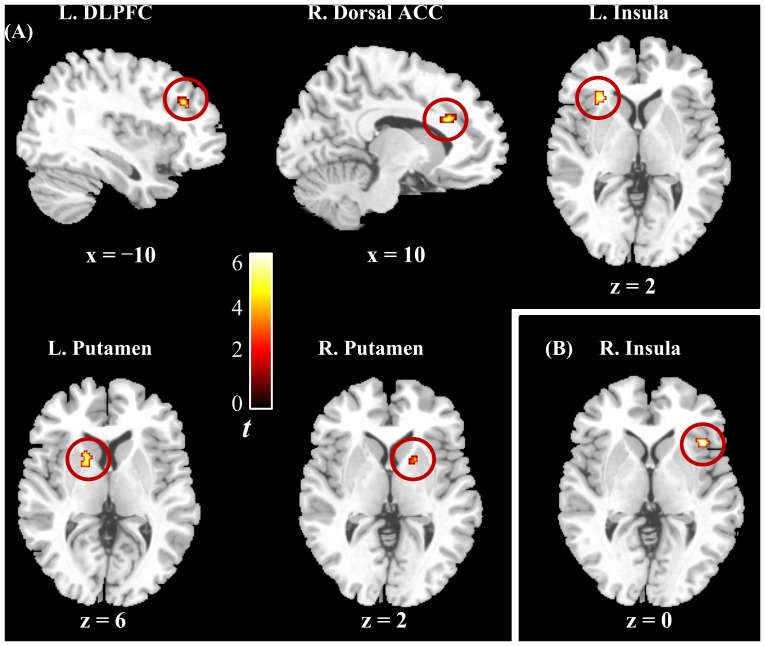
Brain regions showing decreased activities in patients compared with controls. Positive findings were observed only in the ambivalent condition (A) and positive condition (B) L., left; R., right; DLPFC, dorsolateral prefrontal cortex; and ACC, anterior cingulate cortex.

Imaging results of screening two-sample *t*-test in each condition are also described in Table S5 and Table S6. In the group comparison after SVC, compared with controls, patients showed significantly decreased activities in the left DLPFC, right dorsal ACC, left insula, and bilateral putamen for the ambivalent condition, as well as in the right insula for the positive condition (Table 3 and Figure 3), but demonstrated no significantly increased activities. Percent signal changes in these regions (Table S7) showed no significant correlation with negativity bias and positivity offset scores. In addition, no significant group difference was found in the other a priori regions, such as the VLPFC, amygdala, hippocampus, and parahippocampal gyrus.

**Table 3:** Decreased brain activities after small volume correction analysis in patients with schizophrenia compared with healthy controls.

Brain region (Brodmann area)	Side	Voxel size	MNI Coordinates			Z-max	T
			x	y	z		
*For the ambivalent condition*							
DLPFC (46)	Left	41	−38	32	26	3.79	4.49
Dorsal ACC (32)	Right	45	10	28	26	4.16	5.10
Insula (13)	Left	20	−44	16	2	4.53	5.77
Putamen	Right	18	18	6	2	3.50	4.05
	Left	46	−18	10	6	3.86	4.60
*For the positive condition*							
Insula (13)	Right	25	40	20	0	3.85	4.58
*For the negative condition*	No voxel survive threshold						
*For the neutral condition*	No voxel survive threshold						

The threshold was set at a family-wise error-corrected *p*<0.05. MNI, Montreal Neurological Institute; DLPFC, Dorsolateral prefrontal cortex; ACC, Anterior cingulate cortex.

## Discussion

To explore neural representations related to salience attribution during affective asymmetry in schizophrenia, fMRI was performed during the emotion judgment task in patients with schizophrenia and controls. For the ambivalent condition, both groups interpreted the emotional salience of the stimuli as a negative trait, which could be interpreted as ‘negativity bias.’ This result differed from our assumption that patients with schizophrenia would show a reduction in negativity bias, which we demonstrated in our behavioral study [31]. This discrepancy might be due to a problem with the sample size of this study, as a relatively small sample was included in this study; however, this is more likely to stem from differences in experimental design between the two studies. In the previous study, ambivalent stimuli were presented repeatedly within a block, whereas in the current study, they were presented randomly with other types of emotional stimuli. As predictability is known to affect stimulus salience [38], a block design, in which participants can expect a next emotional stimulus, may not evoke significant salience attribution. Therefore, considering that salience attribution was the main focus in this experiment, we presented the stimuli in an event-related design rather than a block design. The current method may increase the mental burden because no order can be predicted. This possibility is supported by the fact that reaction times for almost all conditions were longer in the current study than in the previous study. Consistent with this, another study using the event-related design similar to the one used in the current study found no significant differences in the processing of negativity bias between patients with schizophrenia and controls [11]. This behavioral response may be based on brain neural responses in that brain activity during the processing of emotional content is dependent not only on the type of stimuli, but also the manner in which stimuli are processed [39].

Despite the absence of significant behavioral differences in the ambivalent condition, remarkable group differences in the salience processing triggered by ambivalent stimuli were revealed in the medial prefrontal cortex and inferior frontal gyrus, which exhibited an interaction effect between group and condition. Significantly higher activity in the ambivalent condition than the other conditions was found in controls, but not in patients. The medial prefrontal cortex and inferior frontal gyrus have been proposed to be involved in emotional regulation [40], [41] and response selection process [42], [43]. Because regulation occurs when stimuli induce conflicting appraisals and hence incompatible response tendencies or when goal-directed activity requires suppression of task irrelevant stimulus sources [44], [45], ambivalent stimuli may induce conflicting emotional appraisals and responses that require regulation. In the ambivalent condition, these two regions may need to work hard to regulate ongoing emotional reactions to visual stimuli, which contain conflicting emotions, and to select an appropriate response, whereas this need may be unnecessary in the other conditions. However, patients with schizophrenia did not show these characteristic responses, and these deficits may be related to emotional blunting, which was demonstrated by less positive and negative responses for the positive and negative conditions in this study, respectively. Emotional blunting in schizophrenia may be associated with deficits in emotional regulation. This view is supported by a previous study that showed that dysfunction in the medial prefrontal cortex may be a core of trait anhedonia in schizophrenia [46].

The inferior frontal gyrus receives projections from the orbitofrontal cortex and subcortical areas such as the midbrain and amygdala, which are involved in processing motivational and emotional information [47], [48]. The inferior frontal gyrus functions to integrate cognitive and motivational information to compute behavioral significance, which can be used for elaborate decision-making or to design goal-directed behaviors [49], [50]. In addition, affective asymmetry-related regions such as the DLPFC were found to be decreased in patients with schizophrenia when processing ambivalent stimuli. Given that the DLPFC is involved in integrative processing during co-activation of positivity and negativity [30], this result suggests that patients have deficits in this integrative processing, which could result in inappropriate affective responses. Taken together, despite the intact behavioral response of negativity bias, patients with schizophrenia may not be able to integrate emotional and motivational information during processing of ambivalent stimuli.

The dorsal ACC, insula, and putamen were hypoactive in patients, relative to controls, for the ambivalent condition. These three regions are part of the salience network, which functions to identify the most relevant of several internal and extrapersonal stimuli to guide appropriate behavior [15]. Deficient activities in these regions suggest a dysfunctional salience network in the ambivalent condition. A primary role of the salience network is the integration of sensations, internally generated thoughts, and information concerning goals and plans to allow actions to be initiated or modified [51]. A dysfunctional salience network might also lead to a defect in integration of goals and plans, resulting in the difficulty to initiate activity, which might account for psychomotor poverty syndrome in schizophrenia [51].

Although both groups showed negativity bias in the current design, we were able to confirm our hypothesis of abnormally decreased activity in the affective asymmetry and salience related regions in patients. Compared with controls, patients showed similar negative responses in the ambivalent condition, but decreased negative or positive responses in the univalent conditions, suggesting that an underlying mechanism of negativity bias may be different between the two groups. There is evidence that patients with schizophrenia use a different prefrontal network and strategies in the executive control process for successfully performing a working memory task [52]. Likewise, patients might take a distinct path to obtain negativity bias. One explanation may rely on an accomplishment of negativity bias by a compensatory process for deficient emotional regulations. Functional changes in the DLPFC in patients appeared as decreased activity in BA 46 for the ambivalent condition. In contrast, patients also showed increased activities in BA 8 for the ambivalent, positive, and neutral conditions, which were presented only in the supplementary table, because it was not included as the a priori regions for SVC analysis. Increased activity in an unsuspected region may indicate that this region is working harder to compensate for decreased activity in other regions. Thus, hyperfunction of BA 8 in schizophrenia may occur in order to compensate for hypofunction of the affective asymmetry or salience network.

Meanwhile, patients had significantly lower nPnN response rates than controls for the neutral condition, suggesting that they have a tendency to impart emotional salience to neutral stimuli. While other emotional stimuli have characteristics of primary inducers that automatically and obligatorily elicit emotional responses, neutral stimuli have characteristics of secondary inducers, which are related to “thought,” “memories”, or “imagination” [53]. Therefore, attributing emotional salience to neutral stimuli may be related to the abnormal thought processes of patients with schizophrenia. This behavioral characteristic may be related to previous neuroimaging findings in which patients exhibited inappropriately stronger activations in the amygdala [54] and striatum [55] in response to neutral stimuli, suggesting an aberrant salience attribution in schizophrenia. In this experiment, however, we could not find any significant results in the mesolimbic regions such as the amygdala, hippocampus, and parahippocampal gyrus for the neutral condition in patients. Given a report that patients showed elevated activities in the amygdala even for the baseline condition [56], the absence of significant group differences in the neutral condition might be due to baseline hyperactivity of the mesolimbic regions in schizophrenia.

Several limitations of this study should be noted. First, the sample size was relatively small, which might explain why the expected correlations between regional activities and clinical measures were not found. Second, patients were all taking antipsychotic medication. Although the effects of antipsychotics on emotional responses are known to be negligible [57], psychomotor speed could be influenced by the medication. Third, there was a group difference in the level of education, which could have influenced task performance. To address this, we used years of education as a covariate in behavioral and imaging analyses. Finally, although negativity bias or positivity offset scores were calculated as any portion of responses in the ambivalent and neutral condition, respectively, imaging results were compared across all trials in the corresponding condition. This was inevitable because response rates were highly variable across subjects, and the resultant difference might reflect characteristics of the process for making the response selection, regardless of the response.

In summary, emotional salience attribution during the processing of negativity bias in patients with schizophrenia was associated with multiple prefrontal hypoactivities in the medial prefrontal cortex, inferior frontal gyrus, and DLPFC, which might be related to a deficit in integration of positivity and negativity. The salience network regions, such as the dorsal ACC, insula, and putamen also showed altered activities during the processing of negativity bias in patients, suggesting abnormal emotional salience attribution in schizophrenia. The neural basis of emotional salience attribution during the processing of positivity offset in schizophrenia was not clarified in this experiment in spite of definite behavioral evidence. These regional abnormalities may underlie defective emotional salience attribution in patients with schizophrenia, which likely influences both symptom formation and social dysfunction.

## Supporting Information

Table S1 Images from the International Affective Picture System used in the emotion judgment task.
(DOCX)

Table S2 Group comparison of reaction times.
(XLSX)

Table S3 Raw data of the response rates and reaction times for the emotion judgment task in each participant.
(DOCX)

Table S4 Brain regions showing significant main and interaction effects.
(DOCX)

Table S5 Decreased brain activation across the whole brain in patients with schizophrenia compared with controls (two-sample t-test).
(DOCX)

Table S6 Increased brain activation across whole brain in patients with schizophrenia compared with controls (two-sample t-test).
(DOCX)

Table S7 Percent signal changes in brain regions showing a significant group difference in two-sample *t*-test after small volume correction.
(XLSX)
