# Supplementary material for: Abnormal Neural Processing during Emotional Salience Attribution of Affective Asymmetry in Patients with Schizophrenia
Source: PLoS One. 2014 Mar 11;9(3):e90792. doi: 10.1371/journal.pone.0090792 (PMC3949688; doi:10.1371/journal.pone.0090792)
Supplement: Table S1 — Images from the International Affective Picture System used in the emotion judgment task. (DOCX) [file pone.0090792.s001.docx]

**Table S1.** Images from the International Affective Picture System used in the emotion judgment task

|  | Image code | File name | Valence | Arousal |
| --- | --- | --- | --- | --- |
|  |  |  | (Mean ± SD) | (Mean ± SD) |
| ***Positive image*** | 2540 | Mother | 7.63 ± 1.51 | 3.97 ± 2.33 |
|  | 4599 | Romance | 7.12 ± 1.48 | 5.69 ± 1.94 |
|  | 4626 | Wedding | 7.60 ± 1.66 | 5.78 ± 2.42 |
|  | 4660 | Erotic couple | 7.40 ± 1.36 | 6.58 ± 1.88 |
|  | 5831 | Seagulls | 7.63 ± 1.15 | 4.43 ± 2.49 |
|  | 7502 | Castle | 7.75 ± 1.40 | 5.91 ± 2.31 |
|  | 8200 | Waterskier | 7.54 ± 1.37 | 6.35 ± 1.98 |
|  | 8496 | Waterslide | 7.58 ± 1.63 | 5.79 ± 2.26 |
|  | 8540 | Athletes | 7.48 ± 1.51 | 5.16 ± 2.37 |
|  | Mean |  | 7.53 ± 0.18 | 5.52 ± 0.86 |
|  |  |  |  |  |
| ***Negative image*** | 2800 | Sad child | 1.78 ± 1.14 | 5.49 ± 2.11 |
|  | 3168 | Mutilation | 1.56 ± 1.06 | 6.00 ± 2.46 |
|  | 3170 | Baby tumor | 1.46 ± 1.01 | 7.21 ± 1.99 |
|  | 6250 | Aimed gun | 2.83 ± 1.79 | 6.54 ± 2.61 |
|  | 6313 | Attack | 1.98 ± 1.38 | 6.94 ± 2.23 |
|  | 9253 | Mutilation | 2.00 ± 1.19 | 5.53 ± 2.40 |
|  | 9301 | Toilet | 2.26 ± 1.56 | 5.28 ± 2.46 |
|  | 9440 | Skulls | 3.67 ± 1.86 | 4.55 ± 2.02 |
|  | 9911 | Car accident | 2.30 ± 1.37 | 5.76 ± 2.10 |
|  | Mean |  | 2.20 ± 0.69 | 5.92 ± 0.85 |
|  | | | | |
| ***Neutral image*** | 7009 | Mug | 4.93 ± 1.00 | 3.01 ± 1.97 |
|  | 7010 | Basket | 4.94 ± 1.07 | 1.76 ± 1.48 |
|  | 7025 | Stool | 4.63 ± 1.17 | 2.71 ± 2.20 |
|  | 7036 | Shipyard | 4.88 ± 1.08 | 3.32 ± 2.04 |
|  | 7037 | Trains | 4.81 ± 1.12 | 3.71 ± 2.08 |
|  | 7150 | Umbrella | 4.72 ± 1.00 | 2.61 ± 1.76 |
|  | 7175 | Lamp | 4.87 ± 1.00 | 1.72 ± 1.26 |
|  | 7211 | Clock | 4.81 ± 1.78 | 4.20 ± 2.40 |
|  | 7235 | Chair | 4.96 ± 1.18 | 2.83 ± 2.00 |
|  | Mean |  | 4.84 ± 0.11 | 2.87 ± 0.82 |

SD, standard deviation.
