# Supplementary material for: Abnormal Neural Processing during Emotional Salience Attribution of Affective Asymmetry in Patients with Schizophrenia
Source: PLoS One. 2014 Mar 11;9(3):e90792. doi: 10.1371/journal.pone.0090792 (PMC3949688; doi:10.1371/journal.pone.0090792)
Supplement: Table S3 — Raw data of the response rates and reaction times for the emotion judgment task in each participant. (DOCX) [file pone.0090792.s003.docx]

**Table S2.** Group comparison of reaction times.

| Condition | Response | Reaction time (msec) | | p |
| --- | --- | --- | --- | --- |
|  |  | Schizophrenia (n=15) | Control (n=14) |  |
| **Ambivalent** | Total | 1443.4 (392.2) | 1364.4 (319.8) | 0.56 |
|  | Most frequent (negative) | 1412.0 (330.2) | 1568.0 (400.3) | 0.70 |
| **Positive** | Total | 1452.6 (406.9) | 1354.2 (338.7) | 0.49 |
|  | Most frequent (positive) | 1486.2 (339.6) | 1345.8 (268.2) | 0.96 |
| **Negative** | Total | 1451.0 (401.3) | 1358.4 (342.6) | 0.52 |
|  | Most frequent (negative) | 1211.3 (206.4) | 1163.0 (223.1) | 0.84 |
| **Neutral** | Total | 1449.0 (393.8) | 1356.8 (329.0) | 0.51 |
|  | Most frequent (nPnN) | 1525.0 (354.3) | 1341.2 (299.8) | 0.54 |

Data are given as mean and standard deviation (SD). Group effects are described with years of education as a covariate. “nPnN” means neither positive nor negative.
