# Supplementary material for: Abnormal Neural Processing during Emotional Salience Attribution of Affective Asymmetry in Patients with Schizophrenia
Source: PLoS One. 2014 Mar 11;9(3):e90792. doi: 10.1371/journal.pone.0090792 (PMC3949688; doi:10.1371/journal.pone.0090792)
Supplement: Table S4 — Brain regions showing significant main and interaction effects. (DOCX) [file pone.0090792.s004.docx]

**Table S4.** Brain regions showing significant main and interaction effects.

| Brain region | Side | Voxel | MNI Coordinates | | | *F* |  |
| --- | --- | --- | --- | --- | --- | --- | --- |
| (Brodmann area) |  | size | x | y | z |  |  |
| *Group effect* |  |  |  |  |  |  |  |
| Middle frontal gyrus (8,9,10), DLPFC (9,46), Premotor cortex (6), Primary sensory cortex (1,2,3), Insula (13), Supramarginal gyrus (40), Precuneus (7), Superior, Middle and Inferior occipital gyrus (18,19), ACC (24,32), PCC (23), Caudate, Cerebellum | Both | 72447 | 12 | -76 | -10 | 703.37 |  |
| Middle frontal gyrus (8) | Left | 532 | -24 | 38 | 40 | 178.91 |  |
|  | Right | 214 | 26 | 38 | 40 | 85.33 |  |
| Motor cortex (4) | Left | 47 | -52 | 0 | 44 | 47.98 |  |
| Insula (13) | Left | 67 | -38 | 8 | 16 | 44.55 |  |
| Supramarginal gyrus (40) | Left | 51 | -50 | -36 | 24 | 28.02 |  |
| Angular gyrus (39) | Left | 31 | -58 | -62 | 24 | 28.44 |  |
| Superior temporal gyrus (22) | Right | 356 | 40 | -52 | 10 | 15.94 |  |
| Middle occipital gyrus (19) | Left | 36 | -46 | -76 | 32 | 88.07 |  |
| Caudate | Left | 990 | -16 | -30 | 18 | 69.39 |  |
|  | Left | 81 | -4 | 12 | 16 | 66.84 |  |
|  |  |  |  |  |  |  |  |
| *Condition effect* |  |  |  |  |  |  |  |
| Middle frontal gyrus (8,6) | Left | 134 | -48 | 18 | 44 | 15.90 |  |
| Middle frontal gyrus (8) | Right | 42 | 40 | 26 | 42 | 15.39 |  |
| Superior parietal lobule (7) | Right | 35 | 28 | -68 | 54 | 15.56 |  |
| Supramarginal gyrus (40) | Left | 211 | -56 | -50 | 34 | 19.75 |  |
|  | Right | 180 | 58 | -48 | 38 | 19.08 |  |
| Angular gyrus (39) | Right | 152 | 44 | -64 | 32 | 17.97 |  |
| Transverse temporal gyrus (41) | Left | 83 | -54 | -24 | 14 | 18.59 |  |
| Putamen | Right | 78 | 32 | -6 | 6 | 17.60 |  |
| Caudate | Left | 48 | -34 | -38 | 8 | 12.96 |  |
|  |  |  |  |  |  |  |  |
| *Interaction effect* |  |  |  |  |  |  |  |
| Medial prefrontal cortex (8,6) | Left | 52 | -2 | 34 | 46 | 13.89 |  |
| Inferior frontal gyrus (44) | Left | 43 | -52 | 18 | 16 | 17.73 |  |

The threshold was set at uncorrected p<0.001 with more than 30 voxels. MNI, Montreal Neurological Institute; DLPFC, Dorsolateral prefrontal cortex; ACC, Anterior cingulate cortex; PCC, Posterior cingulate cortex
