# Supplementary material for: Abnormal Neural Processing during Emotional Salience Attribution of Affective Asymmetry in Patients with Schizophrenia
Source: PLoS One. 2014 Mar 11;9(3):e90792. doi: 10.1371/journal.pone.0090792 (PMC3949688; doi:10.1371/journal.pone.0090792)
Supplement: Table S5 — Decreased brain activation across the whole brain in patients with schizophrenia compared with controls (two-sample t-test). (DOCX) [file pone.0090792.s005.docx]

**Table S5.** Decreased brain activation across the whole brain in patients with schizophrenia compared with controls (Two-sample t-test).

| Brain region  (Brodmann area) | Side | Voxel  size | MNI Coordinates | | | Z-max | T |
| --- | --- | --- | --- | --- | --- | --- | --- |
|  |  |  | x | y | z |  |  |
|  |  |  |  |  |  |  |  |
| ***For the ambivalent condition*** | | | | | | | |
| DLPFC (9) | Right | 190 | 50 | 20 | 24 | 4.25 | 5.27 |
| DLPFC (9,46) | Left | 330 | -38 | 30 | 24 | 4.15 | 5.09 |
| Precuneus (7) | Left | 164 | -12 | -72 | 50 | 4.98 | 6.70 |
| Supramarginal gyrus (40) | Left | 627 | -40 | -34 | 36 | 4.20 | 5.18 |
| Superior parietal lobule (7) | Right | 180 | 34 | -54 | 44 | 4.13 | 5.05 |
| Middle occipital gyrus (19) | Left | 401 | -28 | -76 | 24 | 4.49 | 5.69 |
| Dorsal ACC (32) | Right | 49 | 10 | 28 | 26 | 4.16 | 5.10 |
| Insula (13) | Left | 191 | -44 | 16 | 2 | 4.53 | 5.77 |
| Thalamus | Left | 94 | -10 | -12 | -2 | 3.99 | 4.82 |
| Putamen | Left | 104 | -18 | 10 | 6 | 3.86 | 4.60 |
| Cerebellum | Both | 3104 | -8 | -76 | -32 | 6.12 | 4.70 |
|  |  |  |  |  |  |  |  |
| ***For the positive condition*** |  |  |  |  |  |  |  |
| Inferior frontal gyrus (44) | Left | 45 | -46 | 10 | 2 | 3.44 | 3.97 |
| Motor cortex (4) | Left | 175 | -56 | -12 | 32 | 4.43 | 5.58 |
| Middle occipital gyrus (19) | Left | 45 | -32 | -84 | 10 | 4.49 | 5.70 |
|  | Right | 138 | 36 | -74 | 30 | 3.85 | 4.59 |
| Parahippocampal gyrus (35) | Left | 58 | -10 | -34 | -4 | 3.74 | 4.41 |
| Thalamus | Left | 53 | -12 | -14 | 2 | 3.92 | 4.71 |
| Putamen | Left | 103 | -32 | -16 | 2 | 3.89 | 4.65 |
| Insula (13) | Right | 51 | 40 | 20 | 0 | 3.85 | 4.58 |
| Caudate | Right | 64 | 20 | 16 | 10 | 3.70 | 4.35 |
|  |  |  |  |  |  |  |  |
| ***For the negative condition*** |  |  |  |  |  |  |  |
| Motor cortex (4) | Left | 56 | -58 | -10 | 32 | 3.98 | 4.79 |
| Supramarginal gyrus (40) | Left | 109 | -54 | -34 | 42 | 3.89 | 4.65 |
|  |  |  |  |  |  |  |  |
| ***For the neutral condition*** | | | | | | | |
| Caudate | Right | 40 | 24 | 14 | 14 | 3.55 | 4.12 |

MNI, Montreal Neurological Institute; DLPFC, Dorsolateral prefrontal cortex; ACC, Anterior cingulate cortex
