# Supplementary material for: Abnormal Neural Processing during Emotional Salience Attribution of Affective Asymmetry in Patients with Schizophrenia
Source: PLoS One. 2014 Mar 11;9(3):e90792. doi: 10.1371/journal.pone.0090792 (PMC3949688; doi:10.1371/journal.pone.0090792)
Supplement: Table S6 — Increased brain activation across whole brain in patients with schizophrenia compared with controls (two-sample t-test). (DOCX) [file pone.0090792.s006.docx]

**Table S6.** Increased brain activation across whole brain in patients with schizophrenia compared with controls (two-sample t-test).

| Brain region  (Brodmann area) | Side | Voxel  size | MNI Coordinates | | | Z-max | T |
| --- | --- | --- | --- | --- | --- | --- | --- |
|  |  |  | x | y | z |  |  |
|  |  |  |  |  |  |  |  |
| ***For the ambivalent condition*** | | | | | | | |
| DLPFC (8) | Left | 99 | -26 | 38 | 40 | 4.45 | 5.63 |
|  |  |  |  |  |  |  |  |
| ***For the positive condition*** |  |  |  |  |  |  |  |
| DLPFC (8) | Left | 33 | -24 | 38 | 38 | 3.90 | 4.68 |
|  |  |  |  |  |  |  |  |
| ***For the negative condition*** | No voxel survive threshold | | | | | | |
|  |  |  |  |  |  |  |  |
| ***For the neutral condition*** | | | | | | | |
| DLPFC (8) | Left | 48 | -26 | 38 | 40 | 4.26 | 5.29 |

DLPFC, Dorsolateral prefrontal cortex; MNI, Montreal Neurological Institute
